# Characterization of the human gut virome in metabolic and autoimmune diseases

**DOI:** 10.1186/s41232-022-00218-6

**Published:** 2022-11-01

**Authors:** Kosuke Fujimoto, Daichi Miyaoka, Satoshi Uematsu

**Affiliations:** 1 Department of Immunology and Genomics, Graduate School of Medicine, Osaka Metropolitan University, 1-4-3 Asahi-machi, Abeno-ku, Osaka, 545-8585 Japan; 2 Division of Metagenome Medicine, Human Genome Center, The Institute of Medical Science, The University of Tokyo, 4-6-1 Shirokanedai, Minato-ku, Tokyo, 108-8639 Japan; 3 Division of Innate Immune Regulation, International Research and Development Center for Mucosal Vaccines, The Institute of Medical Science, The University of Tokyo, 4-6-1 Shirokanedai, Minato-ku, Tokyo, 108-8639 Japan; 4 Collaborative Research Institute for Innovative Microbiology, The University of Tokyo, 1-1-1 Yayoi, Bunkyo-ku, Tokyo, 113-8657 Japan

**Keywords:** Microbiome, Bacteriome, Virome, Bacteriophage, Dysbiosis, Pathobiont, Metabolic diseases, Autoimmune diseases

## Abstract

The intestinal microbiome is dominated by bacteria and plays a pivotal role in the occurrence and development of disease, including several metabolic and autoimmune disorders. While intestinal viral communities, primarily made up of bacteriophages, are also thought to play a role in disease pathogenesis in the gastrointestinal tract, they have received much less attention than intestinal bacteria. Thus, there is limited information about the relationship between bacteriophages and disease. This review explores a potential role for the intestinal viral microbiome in various metabolic and autoimmune diseases.

## Background

There is a great deal of evidence associating the intestinal bacterial microbiome (bacteriome) with disease pathogenesis. Abnormalities in the human gut microbiota, known as dysbiosis, are linked to a variety of conditions including metabolic disorders, cardiovascular diseases, inflammatory bowel disease (IBD), and autoimmune diseases. Recent studies have shown that trans-kingdom interactions between bacteriomes and viral microbiomes (viromes) are associated with the pathogenesis of pathobiont-mediated diseases [1–5].

It is worth noting that most intestinal viruses are thought to be bacteriophages (phages), which infect and reproduce inside bacteria [6]. Previous studies report that gut phage populations form unique patterns in individuals, independent of genetic factors [6–9]. While viromes are temporally stable in healthy adults [6–9], the virome composition changes in diseases such as IBD, which includes Crohn’s disease and ulcerative colitis [10]. Recent work has shown that the intestinal virome develops in infants along with corresponding changes to the bacteriome [11]. In addition, fecal microbiota transplantation (FMT), which is a recommended treatment regimen for *Clostridioides difficile* infection (CDI) [12], is shown to alter both the bacteriome and the virome [13, 14]. Thus, phages potentially aid bacterial colonization and help to shape bacteriome structure and homeostatic function through host-pathogen interactions [15–19].

There is no universally conserved gene in viruses that is equivalent to the 16S rRNA gene in bacteria or the internal transcribed spacer in fungi, which are used for taxonomic classification [20, 21]. As a result, whole metagenome shotgun sequencing is required to estimate the diversity and taxonomy of intestinal viruses [22], making metagenome research on intestinal phages more challenging than analysis of intestinal bacteria. The vast majority of sequence reads, termed “viral dark matter,” do not align with currently known viral sequences and present a major obstacle to comprehensively defining viromes [23]. This results from a lack of sufficient viral sequence data in public databases because so few intestinal virus analyses have been conducted to date.

Environmental metagenome research, which includes marine virome analysis and intestinal metagenome research, has pioneered technological advances in this field [24–26]. Advances in bioinformatics tools for virome analysis, including assemblers such as IDBA-UD, MEGAHIT, and MetaSPAdes [27–29], viral sequence detection methods such as VirSorter [18], and contig-based classification tools such as vConTACT [30], have demonstrated that effective contig-based viral classification can be performed [18, 19, 30, 31]. Importantly, multiple displacement amplification (MDA), which has been used for most intestinal virome analyses, was shown to be biased owing to preferential amplification of circular single-stranded DNA (ssDNA), likely explaining why the ssDNA phage, Microviridae, was shown to be dominant in the human intestine [10, 25, 32, 33]. Recently, an Adaptase plus Linker Amplification method was developed to enable the composition of ssDNA viruses to be determined without bias [25]. There was clearly a need to acquire genomic and functional information about the intestinal virome and to further characterize the association between intestinal phages and bacteria using more reliable quantitative methods and informatics tools.

A recent study characterized both the intestinal virome and bacteriome in fecal samples from 101 healthy individuals [3]. By examining the genomic sequences of both viromes and bacteriomes, the bacterial hosts of both temperate and virulent intestinal phages could be effectively determined. Newly identified endolysins associated with *Clostridioides difficile* were shown to lyse a high-level toxin-producing *C. difficile* strain and effectively ameliorate acute infection in mice [3]. Thus, comprehensive metagenome analysis can help to understand and prevent microbe-related disorders.

This review summarizes recent advances in the understanding of metabolic disorders, such as metabolic syndrome (MetS), nonalcoholic fatty liver disease (NAFLD), type 2 diabetes mellitus (T2DM), cardiovascular diseases (CVD), and autoimmune diseases, such as type 1 diabetes mellitus (T1DM), rheumatoid arthritis (RA), and systemic lupus erythematosus (SLE), with a particular focus on the role of human intestinal viromes.

### Role of the intestinal virome in metabolic disorders

While it is well established that the intestinal microbiota contributes to the pathogenesis of metabolic disorders such as obesity, MetS, NAFLD, T2DM, and CVD [34], alterations in the gut virome have also been associated with these diseases. For example, the gut virome was shown to change significantly in mouse models of obesity. The gut viromes in the mucosa of Western diet-fed obese mice are enriched with temperate phages of the Caudovirales order, which associate with the Bacilli, Negativicutes, and Bacteroidia classes. Temperate phages from the Bacteroidia class, in particular, encode stress and niche-specific functions that are advantageous to bacterial host adaptation, supporting the potential of gut phages to impact gut microbial function in response to perturbation [35]. In addition, the viral communities of high-fat diet (HFD)-induced obese mice shifted away from the relatively abundant Siphoviridae towards bacteriophages from the Microviridae family. Particular bacteriophage structural genes declined significantly after the transition to HFD, with a conserved loss of integrase genes, suggesting that these altered viral communities are populated by viruses that are more virulent towards host bacteria [36]. Total viral content in leptin-deficient mice feces is also closely linked with metabolic measures such as body weight, fat mass, and fasting blood glucose and is positively correlated with *Firmicutes*, a type of bacteria associated with obesity while being negatively correlated with *Bacteroides* and Bifidobacteria, bacteria associated with leanness [37]. Alterations in the gut virome are thought to have a role in metabolic disorders. Indeed, a study of HFD-induced obese mice showed reduced weight gain and normalized blood glucose tolerance following fecal virome transplant (FVT) from mice with a lean phenotype. FVT significantly changed the bacterial and viral components of the gut microbiota, in addition to the plasma metabolome and expression profiles of obesity and T2DM-associated genes [38]. It was hypothesized that this effect was the result of FVT-induced reshaping of the gut microbiome, warranting further investigation.

Recently, several human studies have sought to characterize the gut virome during various metabolic disorders (Table 1). One study found that obese individuals had lower gut viral richness and diversity and weaker viral-bacterial interactions than lean controls. Eleven viruses, including *Escherichia* phage, *Geobacillus* phage, and *Lactobacillus* phage, were enriched in obese subjects [39]. Another study found higher phage richness and diversity in children with obesity or MetS than in those of normal weight. The phage contigs found at high prevalence in normal-weight individuals were significantly lower in those with obesity and MetS, suggesting that the expansion of specific phages may decrease the concentration of others, and the loss of some phages found at high levels in normal-weight individuals could be associated with obesity and MetS [40]. The dynamics of the gut bacteriophage community after FMT from healthy donors have also been studied in individuals with MetS. Interestingly, the gut virome in patients with better clinical outcomes more closely resembled the viromes of healthy donors [41]. In a study of the gut virome during NAFLD, the liver manifestation of MetS, patients had significantly lower diversity and proportionately fewer phages than control subjects [42]. Lower diversity and bacteriophage number were associated with the severity of NAFLD-associated histologic fibrosis. While the abundance of several *Lactococcus* and *Leuconostoc* phages was inversely correlated with liver fibrosis severity, the abundance of *Lactobacillus* phages correlated positively with fibrosis severity. The abundance of some phages was inversely associated with the abundance of their respective bacterial hosts; however, viral diversity did not always correlate with bacterial diversity, showing that it is difficult to determine what factors are driving lower viral diversity in NAFLD patients.

**Table 1:** Human studies of the gut virome in metabolic disorders

Disease	Study population	References
Obesity	obese subjects (*n*= 128) and lean controls (*n*= 101) in China	[39]
Obesity + MetS	obese (*n*= 10), obese with MetS (*n*= 8) and normal weight children (*n*= 10) in Mexico	[40]
Obesity + MetS	obese subjects with MetS (*n*= 9: self-stool transplant, *n*= 3; healthy-donor-stool transplant recipients, *n*= 6) in the Netherlands	[41]
NAFLD	NAFLD patients (*n*= 73: NAS of 0–4, *n*= 29; NAS of 5–8 or liver cirrhosis, *n*= 44) and controls without liver disease (*n*= 9) in Germany	[42]
T2DM	T2DM patients (*n*= 71) and nondiabetic controls (*n*= 74) in China	[43]
T2DM + Obesity	T2DM patients with obesity (*n*= 74) and lean controls (*n*= 101) in China	[39]
T2DM	T2DM patients (*n*= 17) and nondiabetic controls (*n*= 29) in China	[44]
CVD	coronary heart disease inpatients (*n*= 37) and healthy residents without a history of CVD (*n*= 6) in China	[45]
CVD	atherosclerotic CVD patients (*n*= 218) and healthy controls (*n*= 187) in China	[46]
Hypertension	hypertension patients (*n*= 99), prehypertension (*n*= 56) and healthy controls (*n*= 41) in China	[47]

*Abbreviations*: *MetS* Metabolic syndrome, *NAFLD* Non-alcoholic fatty liver disease, NAS NAFLD Activity Score, *T2DM* Type 2 diabetes mellitus, *CVD* Cardiovascular disease.

Ma et al. first identified seven phage operational taxonomic units (pOTUs) (four Siphoviridae, two Podoviridae, and one *unclassified* family) associated with T2DM patients. However, these T2DM-specific changes in the phageome could not simply be explained as co-variation with their altered bacterial hosts, suggesting that bacteriophages play a complex role in the gut environment that is not limited to interactions with their bacterial hosts [43]. Yang et al. characterized the composition of the gut virome in T2DM patients with obesity and found that these patients had more severe gut viral dysbiosis than those with obesity alone. Although four viral species (*Micromonas pusilla* virus, *Cellulophaga* phage, *Bacteroides* phage, and *Halovirus*) were higher and 13 viral species (*Hokovirus*, *Klosneuvirus*, and *Catovirus*, etc.) were lower in T2DM subjects with obesity than in lean controls, all these differential viral species had the markers of T2DM, indicating that T2DM was a critical and independent contributor to gut viral dysbiosis during obesity. In addition, obese subjects with T2DM showed a significant decrease in the number of phage-bacterial correlations and a higher negative correlation compared with lean controls, suggesting that strong and complex viral-bacterial trans-kingdom correlations of the gut microbiota in lean controls were lost during obesity and T2DM [39]. An increased abundance of both Enterobacteriaceae-specific phages and Enterobacteriaceae is also reported in the enteric microbiome of T2DM patients, which may increase the amount of circulating lipopolysaccharide (LPS) and the systemic inflammatory response, leading to insulin resistance. In addition, the combination of eight selected phages (three *Streptococcus* phages, three Enterobacteriaceae-specific phages, one *Enterococcus* phage, and one *Brochothrix* phage) reliably differentiated T2DM patients from nondiabetic controls, demonstrating a potential use for the phageome as a diagnostic indicator of T2DM [44].

A limited number of case-control studies have been conducted to characterize the gut virome during CVD. Guo et al. first showed that the percentage of Virgaviridae, a family of rod-shaped plant viruses, was higher, and the number of viruses of the family Microviridae was lower in coronary heart disease (CHD) groups than in control groups, indicating that virome composition may be linked to daily living habits and medical therapy for CHD [45]. A large cohort study of individuals with atherosclerotic CVD (ACVD) also demonstrated that several bacteriophages were more or less abundant in the gut virome of individuals with ACVD than in healthy controls. The known hosts for ACVD-enriched bacteriophages mostly included bacteria from the family Enterobacteriaceae or the genus *Streptococcus* [46]. In a study of the gut virome in patients with hypertension, a major modifiable risk factor for CVD, two viral types were predominant among the fecal samples, and the primary bacteriophages for viral-type 1 and viral-type 2 were *Erwinia* phage phiEaH2 and *Lactococcus* phage 1706, respectively [47]. Additionally, certain viruses, including *Cnaphalocrocis medinalis* granulovirus, *Cronobacter* phage CR3, and *Streptococcus* virus phiAbc2, could be used as biomarkers to distinguish healthy individuals from those with hypertension or prehypertension. The viruses were shown to have a superior resolution and discriminatory power for identifying hypertension than bacteria, suggesting that they may be used for early-stage diagnostics.

Although many metabolic disorders are associated with various gut viruses, it is difficult to establish a consistent direction of change in the gut virome and whether these alterations are the cause or result of each disease. As with bacteria in the gut microbiome, additional large-scale longitudinal trials in humans and translational animal studies, especially using FVT, are required to better assess trends and causality. Future research identifying a role for the gut virome in the etiology of metabolic disorders may help to direct virome-based therapeutic approaches such as novel phage therapy.

### Role of the intestinal virome in autoimmune diseases

Recent human studies have investigated the association between the intestinal virome and autoimmune diseases such as T1DM (Table 2). T1DM is characterized by the autoimmune destruction of insulin-producing pancreatic islet beta cells. Most cases of T1DM are preceded by the appearance of islet autoimmunity (IA) that can be serologically confirmed by the presence of at least one autoantibody specific for the pancreatic islets, including insulin (IAA), glutamic acid decarboxylase (GADA), protein tyrosine phosphatase-like insulinoma antigen 2 (IA-2A), and zinc transporter 8 (ZnT8A). While eukaryotic viral infections, such as enterovirus infection, have been proposed as a possible cause of IA and T1DM [54], changes to the gut virome have also been suggested as a potential trigger. The pioneering type 1 diabetes prediction and prevention (DIPP) cohort study of children with IA did not find a significant association between the composition of the gut virome and the development of IA [48]. Indeed, CrAssphage signals showed a possible association between fecal samples from children with early-onset IA followed by T1DM and *Bacteroides dorei*, which has been associated with the risk of IA [55]. However, no significant correlation between the CrAssphage and IA onset has been identified [49].

**Table 2:** Human studies of the intestinal virome in autoimmune diseases

Disease	Study population	References
IA	children with high-risk HLA genotypes who seroconverted (*n*= 19, 18) and matched controls in Finland	[48, 49]
IA	children with high-risk HLA genotypes who seroconverted (*n*= 11) and matched controls in Finland and Estonia	[50]
IA/T1DM	children with high-risk HLA genotypes (*n*= 10: seroconverted, *n*= 6; progressed to T1DM, *n*= 4) and non-seroconverted control individuals (*n*= 8) in Finland and Estonia	[51]
At risk for RA	FDRs of individuals with RA (*n*= 16: with anti-CCP antibodies, *n*= 8; without anti-CCP antibodies, *n*= 8) and healthy matched controls (*n*= 9) in the United States	[52]
RA, SLE, MS	patients with RA (*n*= 111), SLE (*n*= 47), MS (*n*= 29) and healthy controls (*n*= 289) in Japan	[53]

*Abbreviations*: *IA* Islet autoimmunity, *HLA* Human leukocyte antigen, *T1DM* Type 1 diabetes mellitus, *FDR* First-degree relative, *SCCP* Cyclic citrullinated peptide, *RA* Rheumatoid arthritis, *SLE* Systemic lupus erythematosus, *MS* Multiple sclerosis

Zhao et al. found that two major bacteriophage groups, Myoviridae and Podoviridae, had higher diversity and richness in stool samples from control children than in those from children with IA [50]. Amyloid-producing *Escherichia coli* (*E*. *coli*) in the intestine have also been linked to the development of IA and diabetogenic role of *E*. *coli* prophages. An increase in the ratio of *E. coli* phage to *E. coli* was observed prior to bacterial host depletion in children who seroconverted or developed T1DM, suggesting that continual induction of prophages or *E. coli*-harboring prophages may have a fitness advantage in T1DM [51].

While a large number of studies have characterized the gut virome during T1DM, few have characterized the gut virome during systemic autoimmune disease. In a study of individuals at risk for developing rheumatoid arthritis (RA) [52], the composition of the intestinal bacteriophage diverged based on anti-cyclic citrullinated peptide (CCP) autoantibody status, a strong indicator of future RA development. CCP-positive individuals had significantly higher levels of Streptococcaceae, Bacteroidaceae, and Lachnospiraceae phages than healthy controls, indicating the potential of using these phages as biomarkers for preclinical RA. These phages also encoded unique repertoires of auxiliary metabolic genes (AMGs), which modify LPS and other outer membrane glycans on host bacteria, suggesting that they can directly influence the metabolic and immunomodulatory capacity of RA-associated microbiota. Following previous case-control studies of the intestinal bacteriome in Japanese patients with various autoimmune diseases, including RA, systemic lupus erythematosus (SLE), and multiple sclerosis (MS) [56–58], Tomofuji et al. recently revealed disease-associated changes in the intestinal virome [53]. In particular, crAss-like phages, which are a primary component of the healthy gut virome, and particular *Bacteroidetes* and *Firmicutes*, including *Ruminococcus* spp., significantly decreased in the gut of patients with RA and SLE. Podoviridae, which has a symbiotic relationship with *Faecalibacterium*, also declined significantly in the gut of patients with SLE. This evidence suggests that specific phages are associated with autoimmune diseases, possibly by modulating the biological properties of certain bacteria and the host immune system.

Despite great efforts to characterize the gut virome during autoimmune diseases, longitudinal human studies in different populations are needed for a more comprehensive understanding of its role. Novel findings from human studies on the immunomodulatory potential of gut viromes must be confirmed by further investigation using specific T1DM, RA, and SLE animal models.

## Conclusions

The intestinal virome has emerged as a pivotal factor responsible for regulating the pathogenesis of intestinal bacteria-mediated diseases. Because many intestinal phages remain uncharacterized, it is difficult to accurately characterize the intestinal virome. However, this will be overcome by further improvements in metagenomic technology, such as the combination of short-read sequencing and long-read sequencing.

Both genetic and environmental factors have a central role in the pathogenesis of metabolic and autoimmune diseases. Therefore, it would be an interesting future direction to elucidate disease mechanisms by integrated analysis of human genomics and intestinal virome.

Since it is necessary to kill only pathobiont without affecting beneficial bacteria in controlling dysbiosis, the host specificity of the bacteriophage deserves attention for this purpose, as in the regulation of multidrug-resistant bacteria. In addition to bacteria, vast numbers of viruses cohabit the human intestine, and a major fraction of the intestinal virome is composed of bacteriophages, whose hosts are commensal bacteria. Therefore, phage therapy is an alternative treatment for the control of dysbiosis and the elimination of pathobionts. Genomic information about intestinal phages is essential for understanding the intestinal bacteria-phage association [3] and will aid the development of next-generation phage therapies. Thus, it is critically important to expand metagenomic technology and its potential clinical applications.

## Data Availability

Not applicable

## Abbreviations

IBD: Inflammatory bowel disease
FMT: Fecal microbiome transplantation
CDI: *Clostridioides difficile* infection
MDA: Multiple displacement amplification
ssDNA: Single-stranded DNA
MetS: Metabolic syndrome
NAFLD: Nonalcoholic fatty liver disease
CVD: Cardiovascular disease
T1DM: Type 1 diabetes mellitus
T2DM: Type 2 diabetes mellitus
RA: Rheumatoid arthritis
SLE: Systemic lupus erythematosus
HFD: High-fat diet
FVT: Fecal virome transplant
pOTUs: Phage operational taxonomic units
LPS: Lipopolysaccharide
CHD: Coronary heart disease
ACVD: Atherosclerotic cardiovascular disease
IA: Islet autoimmunity
IAA: Insulin autoantibodies
GADA: Glutamic acid decarboxylase antibodies
IA-2A: Tyrosine phosphatase-like insulinoma antigen 2
ZnT8A: Zinc transporter 8 autoantibody
CCP: Cyclic citrullinated peptide
AMGs: Auxiliary metabolic genes
MS: Multiple sclerosis
